# Supplementary material for: Epidemiology of tobacco use in Qatar: Prevalence and its associated factors
Source: PLoS One. 2021 Apr 15;16(4):e0250065. doi: 10.1371/journal.pone.0250065 (PMC8049255; doi:10.1371/journal.pone.0250065)
Supplement: S1 File — (DOCX) [file pone.0250065.s001.docx]

**S1 File.**

**Epidemiology of tobacco use in Qatar: prevalence and its associated factors**

Ahmad AlMulla, ^1^ Ravinder Mamtani^2^, Sohaila Cheema^2^, Patrick Maisonneuve^3^, Jamal Abdullah BaSuhai^1^, Gafar Mahmoud^1^, Silva P. Kouyoumjian^1*^

^1^ *Tobacco Control Center, WHO Collaborative Center, Department of Medicine, Hamad Medical Corporation, Doha, Qatar.*

*^2^ Institute for Population Health, Weill Cornell Medicine-Qatar, Doha, Qatar.*

^3^ *Unit of Clinical Epidemiology, IEO Istituto Europeo di Oncologia IRCSS, Milan, Italy.*

***Correspondence:** Ms. Silva Kouyoumjian, Tobacco Control Center, WHO Collaborative Center, Department of Medicine, Hamad Medical Corporation, P.O. Box 3050, Doha, Qatar. Telephone: + (974) 4025-4858. E-mail: SKouyoumjian@hamad.qa

1. **Methods**

**S1 Fig**. The randomly selected clusters included in the study


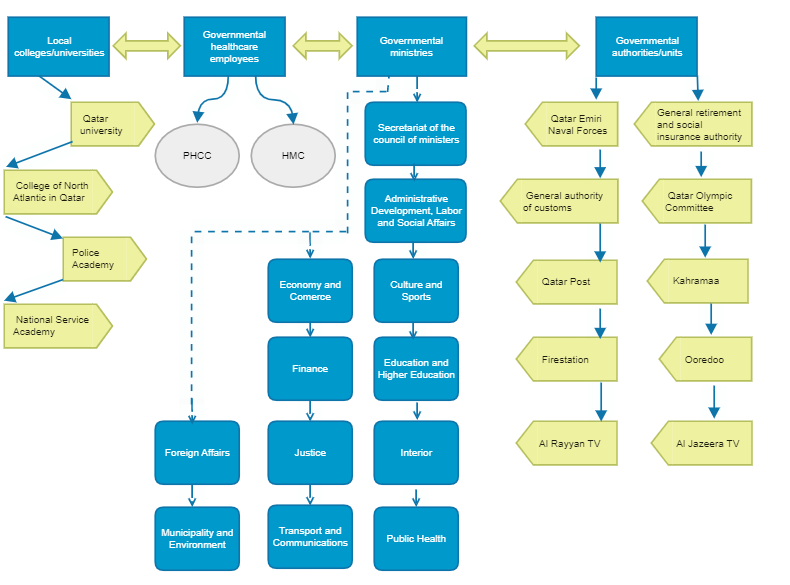


**S1 Appendix**. The survey study in English

**S2 Appendix**. The survey study in Arabic

1. **Results**

**S1 Table**. The total number of comorbidities (n=2453)

| **Comorbidities** | **n** |
| --- | --- |
| As | 222 |
| AsCh | 9 |
| AsChOt | 1 |
| AsEp | 1 |
| AsEpOb | 2 |
| AsHb | 6 |
| AsHbOb | 5 |
| AsHbObCh | 1 |
| AsOb | 36 |
| AsObCh | 4 |
| AsObOt | 3 |
| AsOt | 12 |
| Ch | 239 |
| ChOt | 22 |
| Cv | 41 |
| CvAs | 1 |
| CvAsOb | 2 |
| CvCh | 5 |
| CvDt | 3 |
| CvDtAs | 2 |
| CvDtAsHbCh | 1 |
| CvDtCh | 4 |
| CvDtEpChOt | 1 |
| CvDtEpHbCh | 1 |
| CvDtHb | 4 |
| CvDtHbCh | 4 |
| CvDtHbOb | 3 |
| CvDtHbObCh | 2 |
| CvDtOb | 1 |
| CvDtObCh | 3 |
| CvHb | 5 |
| CvHbCh | 3 |
| CvHbChOt | 1 |
| CvHbOb | 1 |
| CvHbObCh | 1 |
| CvOb | 3 |
| CvOt | 2 |
| Dt | 205 |
| DtAs | 5 |
| DtAsHb | 2 |
| DtAsHbCh | 1 |
| DtAsHbOb | 2 |
| DtAsHbObCh | 2 |
| DtAsOb | 2 |
| DtAsObCh | 2 |
| DtCh | 43 |
| DtEp | 1 |
| DtEpCh | 1 |
| DtEpHbObCh | 1 |
| DtHb | 54 |
| DtHbCh | 25 |
| DtHbOb | 10 |
| DtHbObCh | 11 |
| DtHbObChOt | 2 |
| DtHbObOt | 1 |
| DtHbOt | 5 |
| DtOb | 31 |
| DtObCh | 10 |
| DtObOt | 1 |
| DtOt | 8 |
| Ep | 21 |
| EpCh | 2 |
| EpHb | 1 |
| EpHbCh | 1 |
| EpHbObCh | 1 |
| EpOb | 1 |
| EpObCh | 1 |
| EpObOt | 1 |
| EpOt | 4 |
| Hb | 225 |
| HbCh | 57 |
| HbChOt | 4 |
| HbOb | 41 |
| HbObCh | 15 |
| HbObOt | 2 |
| HbOt | 9 |
| Ob | 479 |
| ObCh | 63 |
| ObChOt | 4 |
| ObOt | 33 |
| Ot | 406 |
| **Grand Total** | **2453** |

Ob=Obesity; Dt=Diabetes; Hb=High blood pressure; As=Asthma; Ch=High Cholesterol levels; Cv=Cardiovascular; Ep=Epilepsy; Ot=Others.

**S2 Table**. The number and percentage of different types of comorbidities (n, %)

|  | **N=6439** | **Prevalence (95% CI)** |
| --- | --- | --- |
| **Comorbidities** |  |  |
| None | 3986 | 61.9% (60.7-63.1) |
| Any | 2453 | 38.1% (36.9 –39.3) |
| **Number of comorbidities** |  |  |
| 1 | 1838 | 28.5% (27.4-29.6) |
| 2 | 458 | 7.1% (6.5-7.7) |
| 3 or more | 157 | 2.4% (2.1-2.8) |
| **Most common comorbidities*** |  |  |
| Obesity | 783 | 12.2% (11.4 –13.0) |
| Diabetes | 454 | 7.1% (6.4 – 7.7) |
| High blood pressure | 510 | 7.9% (7.3 – 8.6) |
| Asthma | 324 | 5.0% (4.5 – 5.6) |
| High cholesterol levels | 548 | 8.5% (7.8 – 9.2) |
| Cardiovascular | 94 | 1.5% (1.2 – 1.8) |
| Epilepsy | 41 | 0.6% (0.4 – 0.8) |
| Others | 522 | 8.1% (7.4 – 8.8) |

*The same subject may be listed in more than 1 category

**S3 Table**. The number of smoking exposures at the living place (n=3195)

| **Exposure of smoking** | **n** |
| --- | --- |
| Br | 1177 |
| BrFr | 15 |
| BrHb | 68 |
| BrHbSo | 2 |
| BrOt | 5 |
| BrRe | 26 |
| BrSi | 18 |
| BrSiHb | 1 |
| BrSiHbSo | 1 |
| BrSiRe | 1 |
| BrSo | 6 |
| Fr | 95 |
| FrRe | 1 |
| Ft | 571 |
| FtBr | 369 |
| FtBrFr | 6 |
| FtBrHb | 28 |
| FtBrHbSo | 1 |
| FtBrOt | 4 |
| FtBrRe | 6 |
| FtBrSi | 7 |
| FtBrSiHb | 1 |
| FtBrSo | 2 |
| FtFr | 11 |
| FtHb | 24 |
| FtHbFr | 1 |
| FtHbRe | 1 |
| FtMt | 25 |
| FtMtBr | 19 |
| FtMtBrRe | 1 |
| FtMtBrSi | 14 |
| FtMtBrSiFr | 1 |
| FtMtBrSiHb | 3 |
| FtMtFr | 1 |
| FtMtHb | 2 |
| FtMtRe | 2 |
| FtMtSi | 8 |
| FtOt | 3 |
| FtRe | 11 |
| FtSi | 8 |
| FtSiHb | 2 |
| FtSo | 7 |
| Hb | 289 |
| HbFr | 1 |
| HbRe | 3 |
| HbSo | 6 |
| Mt | 20 |
| MtBr | 7 |
| MtBrHb | 1 |
| MtBrSi | 11 |
| MtFr | 1 |
| MtHb | 3 |
| MtOt | 1 |
| MtSi | 2 |
| MtSiFr | 1 |
| Ot | 98 |
| Re | 137 |
| Si | 17 |
| SiFr | 1 |
| So | 40 |
| SoOt | 1 |
| **Grand Total** | **3195** |

Ft=Father; Mt=Mother; Br=Brother; Si=Sister; Fr=Friends; Hb=Husband; So=Son; Re=Relatives; Ot=Others

**S4 Table**. The number and percentage of smoking exposures at the living place (n, %)

|  | **N=6798** | **Prevalence (95% CI)** |
| --- | --- | --- |
| **Smokers at home** |  |  |
| None | 3603 | 53.0% (51.8-54.2) |
| Any | 3195 | 47.0% (45.8-48.2) |
| **Number of smokers at home** |  |  |
| 1 | 2444 | 36.0% (34.8-37.1) |
| 2 | 623 | 9.2% (8.5-9.9) |
| 3 or more | 128 | 1.9% (1.6-2.2) |
| **Exposure to smoke at home** |  |  |
| Father | 1139 | 16.8% (15.9-17.6) |
| Mother | 123 | 1.8% (1.5-2.1) |
| Sister* | 97 | 1.4% (1.1-1.7) |
| Brother | 1805 | 26.6% (25.5-27.6) |
| Husband | 438 | 6.4% (5.9-7.0) |
| Relatives/grandparents | 189 | 2.8% (2.4-3.2) |
| Son | 66 | 1.0% (0.7-1.2) |
| Friends/colleagues | 135 | 2.0% (1.7-2.3) |
| Others | 112 | 1.6% (1.3-2.0) |

*Number of individuals exposed to sister(s) smoke at the living place.

**S5 Table.** Prevalence of tobacco use

|  | **N=6904** | **Prevalence (95% CI) %** |
| --- | --- | --- |
| Current | 1741 | 25.2% (24.2-26.2) |
| Daily | 969 | 14.0% (13.2-14.9) |
| Occasional | 772 | 11.2% (10.4-11.9) |
| Ex-user | 828 | 12.0% (11.2-12.8) |
| Daily | 391 | 5.7% (5.1-6.2) |
| Occasional | 437 | 6.3% (5.8-6.9) |
| Not at All | 4335 | 62.8% (61.6-63.9) |
|  |  |  |
| Ever Tried* | 3382 | 50.6% (49.4-51.8) |

* based on 6684 responders with available information

**S6 Table**. The number of different types of current tobacco use by nationality (n, %) smoking refers to tobacco smoking (cigarettes, waterpipe, *medwakh* and cigar)

| Types of tobacco use | Total (n) | Qatari (n) | Non-Qatari (n) |
| --- | --- | --- | --- |
| C | 11 | 3 | 8 |
| CEc | 2 | 2 | - |
| CHtp | 1 | - | 1 |
| Cig | 664 | 224 | 434 |
| CigC | 1 | 1 | - |
| CigCEc | 2 | - | 2 |
| CigEc | 63 | 21 | 41 |
| CigEcHtp | 1 | 1 | - |
| CigHtp | 21 | 13 | 8 |
| CigPsm | 37 | 18 | 17 |
| CigPsmC | 2 | 2 | - |
| CigPsmCSt | 1 | 1 | - |
| CigPsmEc | 10 | 3 | 7 |
| CigPsmHtp | 1 | - | 1 |
| CigPsmSt | 3 | 2 | 1 |
| CigSt | 21 | 14 | 7 |
| CigStEc | 1 | 1 | - |
| CigStHtp | 1 | - | 1 |
| CigWp | 114 | 46 | 65 |
| CigWpC | 5 | 2 | 3 |
| CigWpCEc | 2 | - | 2 |
| CigWpEc | 18 | 10 | 8 |
| CigWpEcHtp | 1 | 1 | - |
| CigWpHtp | 4 | 1 | 3 |
| CigWpPsm | 24 | 13 | 11 |
| CigWpPsmC | 4 | 3 | 1 |
| CigWpPsmCEc | 1 | 1 | - |
| CigWpPsmCEcHtp | 1 | - | 1 |
| CigWpPsmCSt | 3 | 2 | 1 |
| CigWpPsmCStHtp | 1 | 1 | - |
| CigWpPsmEc | 5 | 2 | 3 |
| CigWpPsmSt | 5 | 4 | 1 |
| CigWpPsmStEc | 3 | 3 | - |
| CigWpSt | 8 | 7 | 1 |
| CigWpStEc | 2 | 2 | - |
| CigWpStHtp | 1 | 1 | - |
| CSt | 1 | 1 | - |
| Ec | 31 | 12 | 18 |
| EcHtp | 2 | 2 | - |
| Htp | 5 | 4 | 1 |
| Psm | 50 | 29 | 21 |
| PsmC | 2 | 2 | - |
| PsmEc | 4 | 2 | 2 |
| PsmHtp | 3 | 2 | 1 |
| PsmSt | 3 | 3 | - |
| St | 30 | 18 | 12 |
| StHtp | 1 | 1 | - |
| Wp | 324 | 112 | 205 |
| WpC | 7 | 1 | 6 |
| WpCEc | 1 | - | 1 |
| WpEc | 23 | 11 | 11 |
| WpEcHtp | 1 | - | 1 |
| WpHtp | 2 | 1 | 1 |
| WpPsm | 8 | 6 | 2 |
| WpPsmC | 1 | - | 1 |
| WpPsmSt | 2 | 1 | 1 |
| WpSt | 3 | 3 | - |
| WpStEc | 1 | 1 | - |
| **Grand Total** | **1550** | **617** | **912** |

C=Cigar; Cig=Cigarette; Ec=Electronic cigarette; Htp=Heat-not-burn tobacco products; Psm=Pipe/Sabeel/Medwakh; St=Sweika/Tambak; Wp=Waterpipe

**S7 Table**. The number and percentage of different types of tobacco among current users

| 1. **Cigarette Smoking** | **n** | | 1. **Waterpipe** | | **n** | 1. **Medwakh** | **n** | 1. **Cigar** | | **n** | | 1. **Sweika/Tambak** | **n** |
| --- | --- | --- | --- | --- | --- | --- | --- | --- | --- | --- | --- | --- | --- |
| Cig | 664 | | CigWp | | 114 | CigPsm | 37 | C | | 11 | | CigPsmCSt | 1 |
| CigC | 1 | | CigWpC | | 5 | CigPsmC | 2 | CEc | | 2 | | CigPsmSt | 3 |
| CigCEc | 2 | | CigWpCEc | | 2 | CigPsmCSt | 1 | CHtp | | 1 | | CigSt | 21 |
| CigEc | 63 | | CigWpEc | | 18 | CigPsmEc | 10 | CigC | | 1 | | CigStEc | 1 |
| CigEcHtp | 1 | | CigWpEcHtp | | 1 | CigPsmHtp | 1 | CigCEc | | 2 | | CigStHtp | 1 |
| CigHtp | 21 | | CigWpHtp | | 4 | CigPsmSt | 3 | CigPsmC | | 2 | | CigWpPsmCSt | 3 |
| CigPsm | 37 | | CigWpPsm | | 24 | CigWpPsm | 24 | CigPsmCSt | | 1 | | CigWpPsmCStHtp | 1 |
| CigPsmC | 2 | | CigWpPsmC | | 4 | CigWpPsmC | 4 | CigWpC | | 5 | | CigWpPsmSt | 5 |
| CigPsmCSt | 1 | | CigWpPsmCEc | | 1 | CigWpPsmCEc | 1 | CigWpCEc | | 2 | | CigWpPsmStEc | 3 |
| CigPsmEc | 10 | | CigWpPsmCEcHtp | | 1 | CigWpPsmCEcHtp | 1 | CigWpPsmC | | 4 | | CigWpSt | 8 |
| CigPsmHtp | 1 | | CigWpPsmCSt | | 3 | CigWpPsmCSt | 3 | CigWpPsmCEc | | 1 | | CigWpStEc | 2 |
| CigPsmSt | 3 | | CigWpPsmCStHtp | | 1 | CigWpPsmCStHtp | 1 | CigWpPsmCEcHtp | | 1 | | CigWpStHtp | 1 |
| CigSt | 21 | | CigWpPsmEc | | 5 | CigWpPsmEc | 5 | CigWpPsmCSt | | 3 | | CSt | 1 |
| CigStEc | 1 | | CigWpPsmSt | | 5 | CigWpPsmSt | 5 | CigWpPsmCStHtp | | 1 | | PsmSt | 3 |
| CigStHtp | 1 | | CigWpPsmStEc | | 3 | CigWpPsmStEc | 3 | CSt | | 1 | | St | 30 |
| CigWp | 114 | | CigWpSt | | 8 | Psm | 50 | PsmC | | 2 | | StHtp | 1 |
| CigWpC | 5 | | CigWpStEc | | 2 | PsmC | 2 | WpC | | 7 | | WpPsmSt | 2 |
| CigWpCEc | 2 | | CigWpStHtp | | 1 | PsmEc | 4 | WpCEc | | 1 | | WpSt | 3 |
| CigWpEc | 18 | | Wp | | 324 | PsmHtp | 3 | WpPsmC | | 1 | | WpStEc | 1 |
| CigWpEcHtp | 1 | | WpC | | 7 | PsmSt | 3 | **Grand Total** | | **49** | | **Grand Total** | **91** |
| CigWpPsm | 24 | | WpEc | | 23 | WpPsmC | 1 |  |  |  |  |  |  |
| CigWpPsmC | 4 | | WpEcHtp | | 1 | WpPsmSt | 2 |  |  |  |  |  |  |
| CigWpPsmCEc | 1 | | WpHtp | | 2 | **Grand Total** | **174** |  |  |  |  |  |  |
| CigWpPsmCEcHtp | 1 | | WpPsm | | 8 |  |  |  |  |  |  |  |  |
| CigWpPsmCSt | 3 | | WpPsmC | | 1 |  |  |  |  |  |  |  |  |
| CigWpPsmCStHtp | 1 | | WpPsmSt | | 2 |  |  |  |  |  |  |  |  |
| CigWpPsmEc | 5 | | WpSt | | 3 |  |  |  |  |  |  |  |  |
| CigWpPsmSt | 5 | | WpStEc | | 1 |  |  |  |  |  |  |  |  |
| CigWpPsmStEc | 3 | | **Grand Total** | | **575** |  |  |  |  |  |  |  |  |
| CigWpSt | 8 | |  |  |  |  |  |  |  |  |  |  |  |
| CigWpStEc | 2 | |  |  |  |  |  |  |  |  |  |  |  |
| CigWpStHtp | 1 | |  |  |  |  |  |  |  |  |  |  |  |
| **Grand Total** | **1031** | |  |  |  |  |  |  |  |  |  |  |  |
|  |  | |  |  |  |  |  |  |  |  |  |  |  |
| 1. **Electronic cigarettes** | | **n** | | 1. **Heat-not-burn tobacco products** | | | | | **n** | |  |  |  |
| CEc | | 2 | | CHtp | | | | | 1 | |  |  |  |
| CigCEc | | 2 | | CigEcHtp | | | | | 1 | |  |  |  |
| CigEc | | 63 | | CigHtp | | | | | 21 | |  |  |  |
| CigEcHtp | | 1 | | CigPsmHtp | | | | | 1 | |  |  |  |
| CigPsmEc | | 10 | | CigStHtp | | | | | 1 | |  |  |  |
| CigStEc | | 1 | | CigWpEcHtp | | | | | 1 | |  |  |  |
| CigWpCEc | | 2 | | CigWpHtp | | | | | 4 | |  |  |  |
| CigWpEc | | 18 | | CigWpPsmCEcHtp | | | | | 1 | |  |  |  |
| CigWpEcHtp | | 1 | | CigWpPsmCStHtp | | | | | 1 | |  |  |  |
| CigWpPsmCEc | | 1 | | CigWpStHtp | | | | | 1 | |  |  |  |
| CigWpPsmCEcHtp | | 1 | | EcHtp | | | | | 2 | |  |  |  |
| CigWpPsmEc | | 5 | | Htp | | | | | 5 | |  |  |  |
| CigWpPsmStEc | | 3 | | PsmHtp | | | | | 3 | |  |  |  |
| CigWpStEc | | 2 | | StHtp | | | | | 1 | |  |  |  |
| Ec | | 31 | | WpEcHtp | | | | | 1 | |  |  |  |
| EcHtp | | 2 | | WpHtp | | | | | 2 | |  |  |  |
| PsmEc | | 4 | | **Grand Total** | | | | | **47** | |  |  |  |
| WpCEc | | 1 | |  | | | | |  | |  |  |  |
| WpEc | | 23 | |  |  |  |  |  |  |  |  |  |  |
| WpEcHtp | | 1 | |  |  |  |  |  |  |  |  |  |  |
| WpStEc | | 1 | |  |  |  |  |  |  |  |  |  |  |
| **Grand Total** | | **175** | |  |  |  |  |  |  |  |  |  |  |
|  | |  | |  |  |  |  |  |  |  |  |  |  |

C=Cigar; Cig=Cigarette; Ec=Electronic cigarette; Htp=Heat-not-burn tobacco products; Psm=Pipe/Sabeel/Medwakh; St=Sweika/Tambak; Wp=Waterpipe

**S8 Table**. The number and percentage of different types of tobacco among current Qatari users

| **Cigarette** | n | **Waterpipe** | | **n** | **Medwakh** | | **n** | | **Cigar** | | **n** |
| --- | --- | --- | --- | --- | --- | --- | --- | --- | --- | --- | --- |
| Cig | 224 | CigWp | | 46 | CigPsm | | 18 | | C | | 3 |
| CigC | 1 | CigWpC | | 2 | CigPsmC | | 2 | | CEc | | 2 |
| CigEc | 21 | CigWpEc | | 10 | CigPsmCSt | | 1 | | CigC | | 1 |
| CigEcHtp | 1 | CigWpEcHtp | | 1 | CigPsmEc | | 3 | | CigPsmC | | 2 |
| CigHtp | 13 | CigWpHtp | | 1 | CigPsmSt | | 2 | | CigPsmCSt | | 1 |
| CigPsm | 18 | CigWpPsm | | 13 | CigWpPsm | | 13 | | CigWpC | | 2 |
| CigPsmC | 2 | CigWpPsmC | | 3 | CigWpPsmC | | 3 | | CigWpPsmC | | 3 |
| CigPsmCSt | 1 | CigWpPsmCEc | | 1 | CigWpPsmCEc | | 1 | | CigWpPsmCEc | | 1 |
| CigPsmEc | 3 | CigWpPsmCSt | | 2 | CigWpPsmCSt | | 2 | | CigWpPsmCSt | | 2 |
| CigPsmSt | 2 | CigWpPsmCStHtp | | 1 | CigWpPsmCStHtp | | 1 | | CigWpPsmCStHtp | | 1 |
| CigSt | 14 | CigWpPsmEc | | 2 | CigWpPsmEc | | 2 | | CSt | | 1 |
| CigStEc | 1 | CigWpPsmSt | | 4 | CigWpPsmSt | | 4 | | PsmC | | 2 |
| CigWp | 46 | CigWpPsmStEc | | 3 | CigWpPsmStEc | | 3 | | WpC | | 1 |
| CigWpC | 2 | CigWpSt | | 7 | Psm | | 29 | | **Grand Total** | | **22** |
| CigWpEc | 10 | CigWpStEc | | 2 | PsmC | | 2 | |  | |  |
| CigWpEcHtp | 1 | CigWpStHtp | | 1 | PsmEc | | 2 | |  |  |  |
| CigWpHtp | 1 | Wp | | 112 | PsmHtp | | 2 | |  |  |  |
| CigWpPsm | 13 | WpC | | 1 | PsmSt | | 3 | |  |  |  |
| CigWpPsmC | 3 | WpEc | | 11 | WpPsm | | 6 | |  |  |  |
| CigWpPsmCEc | 1 | WpHtp | | 1 | WpPsmSt | | 1 | |  |  |  |
| CigWpPsmCSt | 2 | WpPsm | | 6 | **Grand Total** | | **100** | |  |  |  |
| CigWpPsmCStHtp | 1 | WpPsmSt | | 1 |  |  |  |  |  |  |  |
| CigWpPsmEc | 2 | WpSt | | 3 |  |  |  |  |  |  |  |
| CigWpPsmSt | 4 | WpStEc | | 1 |  |  |  |  |  |  |  |
| CigWpPsmStEc | 3 | **Grand Total** | | **235** |  |  |  |  |  |  |  |
| CigWpSt | 7 |  |  |  |  |  |  |  |  |  |  |
| CigWpStEc | 2 |  |  |  |  |  |  |  |  |  |  |
| CigWpStHtp | 1 |  |  |  |  |  |  |  |  |  |  |
| **Grand Total** | **400** |  |  |  |  |  |  |  |  |  |  |
|  |  |  |  |  |  |  |  |  |  |  |  |
| **Sweika** | **n** | **Ecigarettes** | **n** | | | **Htp** | | **n** | |  |  |
| CigPsmCSt | 1 | CEc | 2 | | | CigEcHtp | | 1 | |  |  |
| CigPsmSt | 2 | CigEc | 21 | | | CigHtp | | 13 | |  |  |
| CigSt | 14 | CigEcHtp | 1 | | | CigWpEcHtp | | 1 | |  |  |
| CigStEc | 1 | CigPsmEc | 3 | | | CigWpHtp | | 1 | |  |  |
| CigWpPsmCSt | 2 | CigStEc | 1 | | | CigWpPsmCStHtp | | 1 | |  |  |
| CigWpPsmCStHtp | 1 | CigWpEc | 10 | | | CigWpStHtp | | 1 | |  |  |
| CigWpPsmSt | 4 | CigWpEcHtp | 1 | | | EcHtp | | 2 | |  |  |
| CigWpPsmStEc | 3 | CigWpPsmCEc | 1 | | | Htp | | 4 | |  |  |
| CigWpSt | 7 | CigWpPsmEc | 2 | | | PsmHtp | | 2 | |  |  |
| CigWpStEc | 2 | CigWpPsmStEc | 3 | | | StHtp | | 1 | |  |  |
| CigWpStHtp | 1 | CigWpStEc | 2 | | | WpHtp | | 1 | |  |  |
| CSt | 1 | Ec | 12 | | | **Grand Total** | | **28** | |  |  |
| PsmSt | 3 | EcHtp | 2 | | |  |  |  |  |  |  |
| St | 18 | PsmEc | 2 | | |  |  |  |  |  |  |
| StHtp | 1 | WpEc | 11 | | |  |  |  |  |  |  |
| WpPsmSt | 1 | WpStEc | 1 | | |  |  |  |  |  |  |
| WpSt | 3 | **Grand Total** | **75** | | |  |  |  |  |  |  |
| WpStEc | 1 |  |  |  |  |  |  |  |  |  |  |
| **Grand Total** | **66** |  |  |  |  |  |  |  |  |  |  |

C=Cigar; Cig=Cigarette; Ec=Electronic cigarette; Htp=Heat-not-burn tobacco products; Psm=Pipe/Sabeel/Medwakh; St=Sweika/Tambak; Wp=Waterpipe

**S9 Table**. The number and percentage of different types of tobacco among current non-Qatari users

| **Cigarette** | **n** | **Wp** | **n** | **Medwakh** | **n** | **Cigar** | **n** |
| --- | --- | --- | --- | --- | --- | --- | --- |
| Cig | 434 | CigWp | 65 | CigPsm | 17 | C | 8 |
| CigCEc | 2 | CigWpC | 3 | CigPsmEc | 7 | CHtp | 1 |
| CigEc | 41 | CigWpCEc | 2 | CigPsmHtp | 1 | CigCEc | 2 |
| CigHtp | 8 | CigWpEc | 8 | CigPsmSt | 1 | CigWpC | 3 |
| CigPsm | 17 | CigWpHtp | 3 | CigWpPsm | 11 | CigWpCEc | 2 |
| CigPsmEc | 7 | CigWpPsm | 11 | CigWpPsmC | 1 | CigWpPsmC | 1 |
| CigPsmHtp | 1 | CigWpPsmC | 1 | CigWpPsmCEcHtp | 1 | CigWpPsmCEcHtp | 1 |
| CigPsmSt | 1 | CigWpPsmCEcHtp | 1 | CigWpPsmCSt | 1 | CigWpPsmCSt | 1 |
| CigSt | 7 | CigWpPsmCSt | 1 | CigWpPsmEc | 3 | WpC | 6 |
| CigStHtp | 1 | CigWpPsmEc | 3 | CigWpPsmSt | 1 | WpCEc | 1 |
| CigWp | 65 | CigWpPsmSt | 1 | Psm | 21 | WpPsmC | 1 |
| CigWpC | 3 | CigWpSt | 1 | PsmEc | 2 | **Grand Total** | **27** |
| CigWpCEc | 2 | Wp | 205 | PsmHtp | 1 |  |  |
| CigWpEc | 8 | WpC | 6 | WpPsm | 2 |  |  |
| CigWpHtp | 3 | WpCEc | 1 | WpPsmC | 1 |  |  |
| CigWpPsm | 11 | WpEc | 11 | WpPsmSt | 1 |  |  |
| CigWpPsmC | 1 | WpEcHtp | 1 | **Grand Total** | **72** |  |  |
| CigWpPsmCEcHtp | 1 | WpHtp | 1 |  |  |  |  |
| CigWpPsmCSt | 1 | WpPsm | 2 |  |  |  |  |
| CigWpPsmEc | 3 | WpPsmC | 1 |  |  |  |  |
| CigWpPsmSt | 1 | WpPsmSt | 1 |  |  |  |  |
| CigWpSt | 1 | **Grand Total** | **329** |  |  |  |  |
| **Grand Total** | **619** |  |  |  |  |  |  |

| **Swieka** | **n** | **E-cigarettes** | **n** | **Htp** | **n** |
| --- | --- | --- | --- | --- | --- |
| CigPsmSt | 1 | CigCEc | 2 | CHtp | 1 |
| CigSt | 7 | CigEc | 41 | CigHtp | 8 |
| CigStHtp | 1 | CigPsmEc | 7 | CigPsmHtp | 1 |
| CigWpPsmCSt | 1 | CigWpCEc | 2 | CigStHtp | 1 |
| CigWpPsmSt | 1 | CigWpEc | 8 | CigWpHtp | 3 |
| CigWpSt | 1 | CigWpPsmCEcHtp | 1 | CigWpPsmCEcHtp | 1 |
| St | 12 | CigWpPsmEc | 3 | Htp | 1 |
| WpPsmSt | 1 | Ec | 18 | PsmHtp | 1 |
| **Grand Total** | **25** | PsmEc | 2 | WpEcHtp | 1 |
|  |  | WpCEc | 1 | WpHtp | 1 |
|  |  | WpEc | 11 | **Grand Total** | **19** |
|  |  | WpEcHtp | 1 |  |  |
|  |  | **Grand Total** | **97** |  |  |

C=Cigar; Cig=Cigarette; Ec=Electronic cigarette; Htp=Heat-not-burn tobacco products; Psm=Pipe/Sabeel/Medwakh; St=Sweika/Tambak; Wp=Waterpipe

**S10 Table.** Percentage distribution of cigarette brands among current Qatari and Non-Qataris by selected demographic characteristics

|  | Cigarette brands purchased* | | | | | | | |
| --- | --- | --- | --- | --- | --- | --- | --- | --- |
|  | Marlboro | Winston | Kent | Dunhill | Davidoff | Parliament | Philip Morris | Others |
| Total N=1008 | 431 (42.8%) | 42 (4.2%) | 13 (1.3%) | 106 (10.5%) | 122 (12.1%) | 156 (15.5%) | 88 (8.7%) | 80 (7.9%) |
| Nationality |  |  |  |  |  |  |  |  |
| Qatar (N=386) | 161 (41.7%) | 3 (0.8%) | 2 (0.5%) | 49 (12.7%) | 48 (12.4%) | 104 (26.9%) | 17 (4.4%) | 6 (1.6%) |
| Non-Qatari (N=616) | 266 (43.2%) | 39 (6.3%) | 11 (1.8%) | 57 (9.3%) | 73 (11.9%) | 52 (8.4%) | 70 (11.4%) | 74 (12.0%) |
| Qatari |  |  |  |  |  |  |  |  |
| 18-24 | 42 (29.4%) | - | - | 13 (9.1%) | 12 (8.4%) | 68 (47.6%) | 9 (6.3%) | - |
| 25-34 | 49 (45.4%) | 1 (0.9%) | 2 (1.9%) | 13 (12.0%) | 16 (14.8%) | 21 (19.4%) | 5 (4.6%) | 3 (2.8%) |
| 35-44 | 43 (50.6%) | 2 (2.4%) | - | 16 (18.8%) | 11 (12.9%) | 9 (10.6%) | 3 (3.5%) | 1 (1.2%) |
| 45-54 | 24 (57.1%) | - | - | 5 (11.9%) | 7 (16.7%) | 5 (11.9%) | - | 2 (4.8%) |
| 55+ | 3 (37.5%) | - | - | 2 (25.0%) | 2 (25.0%) | 1 (12.5%) | - | - |
| Non-Qatari |  |  |  |  |  |  |  |  |
| 18-24 | 49 (45.4) | 7 (6.5%) | 5 (4.6%) | 8 (7.4%) | 6 (5.6%) | 23 (21.3%) | 16 (14.8%) | 11 (10.2%) |
| 25-34 | 86 (43.0%) | 14 (7.0%) | 1 (0.5%) | 17 (8.5%) | 26 (13.0%) | 13 (6.5%) | 24 (12.0%) | 23 (11.5%) |
| 35-44 | 88 (43.3%) | 10 (4.9%) | 4 (2.0%) | 22 (10.8%) | 24 (11.8%) | 13 (6.4%) | 17 (8.4%) | 29 (14.3%) |
| 45-54 | 34 (39.5%) | 7 (8.1%) | 1 (1.2%) | 10 (11.6%) | 13 (15.1%) | 2 (2.3%) | 9 (10.5%) | 11 (12.8%) |
| 55+ | 9 (47.4%) | 1 (5.3%) | - | - | 4 (21.1%) | 1 (5.3%) | 4 (21.1%) | - |

*The same subject may be listed in more than 1 category
