# Supplementary material for: Epidemiology of tobacco use in Qatar: Prevalence and its associated factors
Source: PLoS One. 2021 Apr 15;16(4):e0250065. doi: 10.1371/journal.pone.0250065 (PMC8049255; doi:10.1371/journal.pone.0250065)
Supplement: Attachment — Submitted filename: Reply to Reviewers_PlosOneR1.docx [file pone.0250065.s002.docx]

**Epidemiology of tobacco use in Qatar: prevalence and its associated factors**

Ahmad AlMulla, ^1^ Ravinder Mamtani^2^, Sohaila Cheema^2^, Patrick Maisonneuve^3^, Jamal Abdullah BaSuhai^1^, Gafar Mahmoud^1^, Silva P. Kouyoumjian^1*^

^1^ *Tobacco Control Center, WHO Collaborative Center, Department of Medicine, Hamad Medical Corporation, Doha, Qatar.*

*^2^ Institute for Population Health, Weill Cornell Medicine-Qatar, Doha, Qatar.*

^3^ *Unit of Clinical Epidemiology, IEO Istituto Europeo di Oncologia IRCSS, Milan, Italy.*

REPLY TO EDITOR AND REVIEWER COMMENTS

All authors would like to thank the editor and the reviewer for assessing our work and for providing us with the constructive feedback that will further improve the article’s quality and its readability. Please find below a point-by-point response addressing the editor/reviewer comments. We have also incorporated these suggestions in the revised manuscript as noted below.

Note: All references to the revised manuscript pertain to the marked copy of these files including changes implemented through “track changes”.

**Editorial requests:**

*The reviewer was correct in pointing out the relatively little additional information added by this study to advance our understanding of tobacco use in Qatar. Although Plos One evaluates studies based on their methodological soundness, method-wise accuracy and rigor are also considered an important value to be added by research. I suggest revise the current study by highlighting how the study has a rigorous study design and employed solid method to assess tobacco use in Qatar.*

Response: We would like to thank the Editor for this suggestion. We have now included this suggestion in the manuscript to highlight the rigorous study design and structured approach to assess tobacco use in Qatar:

The study’s research design and implementation approach were based on a systematic process with due attention given to reduce bias at each stage of the study. The field work was exemplary across all sample recruitment sites to ensure that the results were replicable and valid to support future decision-making for tobacco control. (Materials and Methods, 2^nd^ paragraph, page 5-6).

The survey instrument was carefully administered with attention to protocol adherence by trained staff to guarantee a unified procedure. (Materials and Methods, 3^rd^ paragraph, page 8).

We have revised Table 3 (page 28), which we believe is more readable and informative than before. Accordingly, we have revised the percentages in the text as well:

1. Of the 1550 tobacco users, 42.8% were cigarette smokers, 20.9% waterpipe, 3.2% *medwakh* (Arabic traditional pipe) and 0.7% cigar. Moreover, 1.9% reported smokeless tobacco use (*sweika*), 2.0% reported electronic cigarette use, and 0.3% heat-not-burn tobacco use. (Abstract, page 2)
2. Table 3 describes the number and percentage of different types of tobacco among current users by nationality. The numbers of different types of tobacco use are not exclusive and further details are found in S6-S9 Tables. Of the current tobacco users (n=1550), 42.8% were cigarette smokers, 20.9% waterpipe, 3.2% *medwakh* (Arabic traditional pipe) and 0.7% cigar smokers. Moreover, 1.9% reported smokeless tobacco use (*sweika*), 2.0% electronic cigarette use, 0.3% heat-not-burn tobacco use and 28.1% were using more than one type of tobacco product (Table 3). (Results, 2^nd^ paragraph, page 11)
3. Among Qatari current tobacco users (n=617), 36.3% were cigarette smokers, followed by waterpipe smoking (18.2%), *medwakh* smoking (4.7%) and cigar smoking (0.5%). Moreover, 2.9% reported smokeless tobacco use (*sweika*), 1.9% electronic cigarette use, 0.6% heat-not-burn tobacco use and 34.8% were using more than one type of tobacco product (Table 3). (Results, 3^rd^ paragraph, page 11)
4. Among non-Qatari tobacco users (n=912), cigarette smoking was the highest (47.6%), followed by waterpipe smoking (22.5%), *medwakh* smoking (2.3%) and cigar smoking (0.9%). Moreover, 1.3% reported smokeless tobacco use (*sweika*), electronic cigarette use (2.0%), and 0.1% heat-not-burn tobacco use, and 23.4% were using more than one type of tobacco product (Table 3). (Results, 1^st^ paragraph, page 12)
5. The most commonly smoked tobacco product in our study was cigarette (42.8%) and waterpipe (20.9%). (Discussion, last paragraph, page 15)
6. So far there has been no population-based survey reporting the use of *medwakh* in Qatar. In our study, *medwakh* smoking only was more prevalent among Qatari users compared to non-Qatari users (4.7% vs. 2.3%). These rates are lower than the regional rates (30-34), where in United Arab Emirates it is ranked as the second most common form of tobacco (35). Comparable low rates of smokeless tobacco use only was evident among Qatari and non-Qatari tobacco users (2.9% vs. 1.3%), much lower than the limited data found in the published literature (36, 37). (Discussion, 1^st^ paragraph, page 16)
7. Electronic cigarette use only was similar among Qatari tobacco users and non-Qatari tobacco users (1.9% vs. 2.0%). As per the Ministry of Public Health revised law in 2016, the selling, distribution or possession of large number of electronic cigarettes is illegal in Qatar. Despite this, there seems to be an increasing trend in the country in comparison to the results of GATS 2013 (11). (Discussion, 2^nd^ paragraph, page 16)
8. A typo error in Table 2 has been corrected as well. (Table 2, page 26)

1. Please ensure that your manuscript meets PLOS ONE's style requirements, including those for file naming. The PLOS ONE style templates can be found at

https://journals.plos.org/plosone/s/file?id=wjVg/PLOSOne_formatting_sample_main_body.pdf and

https://journals.plos.org/plosone/s/file?id=ba62/PLOSOne_formatting_sample_title_authors_affiliations.pdf

Response: We confirm that the manuscript meets PLOS ONE’s style requirements including those for file naming.

2. Please ensure that you include a title page within your main document. We do appreciate that you have a title page document uploaded as a separate file, however, as per our author guidelines (http://journals.plos.org/plosone/s/submission-guidelines#loc-title-page) we do require this to be part of the manuscript file itself and not uploaded separately.
Could you therefore please include the title page into the beginning of your manuscript file itself, listing all authors and affiliations.

Response: As per the suggestion, a title page has been included into the beginning of the manuscript file itself, listing all the authors and their affiliations.

3. Please include additional information regarding the survey or questionnaire used in the study and ensure that you have provided sufficient details that others could replicate the analyses. For instance, if you developed a questionnaire as part of this study and it is not under a copyright more restrictive than CC-BY, please include a copy, in both the original language and English, as Supporting Information.

Response: Thank you for this comment. The survey tool is available in Arabic and English and is provided as Supporting Information (S1 Appendix and S2 Appendix of SI). (Materials and Methods, page 7, 2^nd^ paragraph).

4.Please include your tables as part of your main manuscript and remove the individual files. Please note that supplementary tables (should remain/ be uploaded) as separate "supporting information" files.

Response: The tables have now been included as part of the main manuscript. (Main document_marked copy, pages 25-30)

5. Please include captions for your Supporting Information files at the end of your manuscript, and update any in-text citations to match accordingly. Please see our Supporting Information guidelines for more information: [http://journals.plos.org/plosone/s/supporting-information](about:blank).

Response: Captions for the Supporting Information files have been included at the end of the manuscript on page 23 and 24 according to PLOS ONE guidelines.

6. Your ethics statement should only appear in the Methods section of your manuscript. If your ethics statement is written in any section besides the Methods, please move it to the Methods section and delete it from any other section. Please ensure that your ethics statement is included in your manuscript, as the ethics statement entered into the online submission form will not be published alongside your manuscript.

Response: The ethics statement i.e “The study protocol was approved by the Institutional Review Board, Medical Research Center, Hamad Medical Corporation, Qatar” now appears only in the Methods section of the manuscript. (Materials and Methods, page 6, 1^st^ paragraph).

**Reviewers' comments to the author:**

This study examined the prevalence of tobacco use and the associated factors among a population-based sample of adults 18 years and above in Qatar. Its sampling methodology, measurement of the variables, and statistical analyses are appropriate. The critical issue is the little insightful information provided to the scholarship.

Response: We thank the reviewer for this comment. Our study now provides insightful information for scholarship as follows (Discussion, 2^nd^ paragraph, page 17 and 1^st^ paragraph, page 18):

The prevalence of tobacco smoking in Qatar has reduced by 15.2% between 2000 and 2019 due to the measures implemented by the country. Our study strengthens the previously limited tobacco evidence available for Qatar. Recommendations and way forward are also discussed.

1) Moreover, the tobacco industry aggressively targets adults with variant tobacco product promotions. There is an increasing trend for dual, poly-tobacco, and nonconventional tobacco product use. Our study provides new insights pertaining to the use of specific tobacco product patterns in Qatar. This has not been previously explored in detail, not even in the recent 2013 GATS survey.
2) Our study findings enhance the evidence base needed to inform health-related programming efforts and other community awareness initiatives needed in Qatar.
3) The study findings can also be used to inform new policy recommendations and offer novel interventions relevant to the local context of Qatar. The rising incidence of waterpipe smoking and multi-tobacco use must be taken into consideration when designing tobacco control programs.
4) To better understand the complex issues surrounding tobacco use in Qatar, further innovative research using rigorous methodology is needed to generate additional data and guide the development of evidence based smoking cessation programs. Also, intervention research is critically needed to determine best practices for reducing tobacco use.
